# Antimicrobial and Prebiotic Activity of Lactoferrin in the Female Reproductive Tract: A Comprehensive Review

**DOI:** 10.3390/biomedicines9121940

**Published:** 2021-12-17

**Authors:** Jolanta Artym, Michał Zimecki

**Affiliations:** Department of Experimental Therapy, Hirszfeld Institute of Immunology and Experimental Therapy, Polish Academy of Sciences, R. Weigla 12 Str., 53-112 Wrocław, Poland; jolanta.artym@hirszfeld.pl

**Keywords:** lactoferrin, probiotics, prebiotic activity, antimicrobial activity, vaginal microbiota

## Abstract

Women’s intimate health depends on several factors, such as age, diet, coexisting metabolic disorders, hormonal equilibrium, sexual activity, drug intake, contraception, surgery, and personal hygiene. These factors may affect the homeostasis of the internal environment of the genital tract: the vulva, vagina and cervix. This equilibrium is dependent on strict and complex mutual interactions between epithelial cells, immunocompetent cells and microorganisms residing in this environment. The microbiota of the genital tract in healthy women is dominated by several species of symbiotic bacteria of the *Lactobacillus* genus. The bacteria inhibit the growth of pathogenic microorganisms and inflammatory processes by virtue of direct and multidirectional antimicrobial action and, indirectly, by the modulation of immune system activity. For the homeostasis of the genital tract ecosystem, antimicrobial and anti-inflammatory peptides, as well as proteins secreted by mucus cells into the cervicovaginal fluid, have a fundamental significance. Of these, a multifunctional protein known as lactoferrin (LF) is one of the most important since it bridges innate and acquired immunity. Among its numerous properties, particular attention should be paid to prebiotic activity, i.e., exerting a beneficial action on symbiotic microbiota of the gastrointestinal and genital tract. Such activity of LF is associated with the inhibition of bacterial and fungal infections in the genital tract and their consequences, such as endometritis, pelvic inflammation, urinary tract infections, miscarriage, premature delivery, and infection of the fetus and newborns. The aim of this article is to review the results of laboratory as well as clinical trials, confirming the prebiotic action of LF on the microbiota of the lower genital tract.

## 1. Introduction

**Figure 1:**
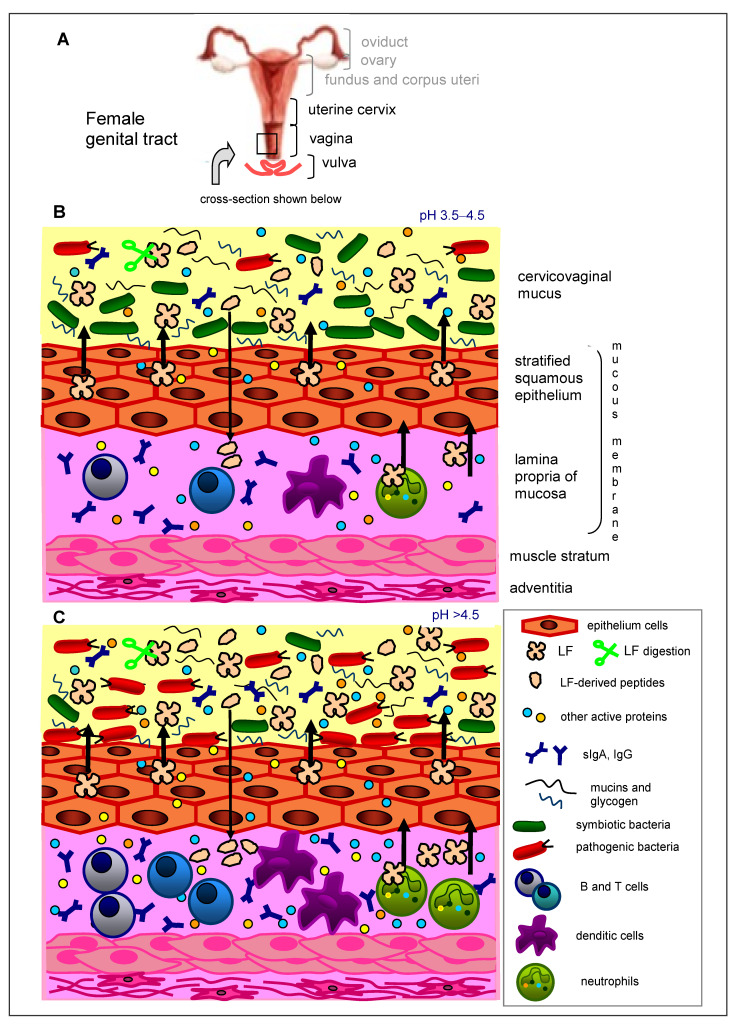
The female genital system with the organs of its lower part (genital tract) (**A**) and the elements of innate immunity in the healthy (**B**) and infected (**C**) genital tract. (**B**) Healthy vaginal and cervical epithelia are protected by a layer of cervicovaginal mucus. Protection against pathogenic viruses, bacteria, fungi and parasites is secured by immune cells (mainly neutrophils, dendritic cells, NK cells and lymphocytes) in the lamina propria of the mucous membrane. The epithelial cells secrete polysaccharides (mucins and glycogen), and the epithelial and immune cells secrete antimicrobial peptides (as LF, SLPI, SP-A and lysozyme), cytokines/chemokines (as IL-1, IL-6, IL-8), and acute phase proteins (such as serum amyloid A and haptoglobin). In the mucus, numerous symbiotic bacteria reside (mainly several genera from *Lactobacillus* spp.) that metabolize glycogen into lactic acid, which renders the genital tract environment acidic (~pH 3.5–4.5). The immune cells and symbiotic microbiota cooperate to prevent microbial invasion of the uterus and vagina. (**C**) During bacterial vaginosis/vaginitis, the number of pathogenic bacteria increases and that of symbiotic bacteria decreases; these perturbations of the vaginal microbiota lead to increased vaginal pH > 4.5, as well as concomitant levels of immune cells and antimicrobial proteins, proinflammatory cytokines and acute-phase proteins.

The genital tract encompasses the uterine cervix, vagina, and vulva (Figure 1A). Its main role is to accept spermatozoa during insemination, as well as to expel the fetus and placenta during delivery and the exfoliated endometrium at the time of menstruation. Due to its direct contact with the external environment, the genital tract is at risk of colonization by pathogenic microorganisms, which may evoke inflammation and infection. Protection against pathogens is ensured by closely collaborating epithelial cells lining the genital tract, residing local immune cells, and microbial cells, which abundantly colonize this environment. A proper ecosystem of the genital tract plays a key role in protecting against infection and inflammation via many different mechanisms. First investigations into microorganisms residing in the vagina were conducted almost 130 years ago when Albert Döderlein (1860–1941) discovered and described anaerobic, curved rods producing lactic acid in the patient’s vagina [1]. During subsequent years, thanks to the application of cultures on agar plates, numerous microorganisms residing in the vagina have been identified and described. Nevertheless, by the time new, culture-independent methods of molecular biology and genetic engineering were applied, it was not possible to investigate them fully. New methods allowed researchers to discover difficult-to-culture anaerobic microorganisms. Knowledge of the ecosystem composition of the genital tract, and its complicated interrelationships, enabled recognition of the etiology of its infectious and inflammatory diseases and eventually allowed new, efficacious drugs to be worked up and applied in clinical practice. We know now that the restoration of the homeostasis of this complex ecosystem is crucial in the therapy of infections and inflammation of the genital tract. This goal can be achieved through the application of probiotic bacteria that assist the growth of beneficial vaginal microorganisms. An additional advantage may be gained by the application of prebiotics targeted at strengthening the condition of used probiotics and resident microorganisms. 

Our diet is a valuable source of probiotics and prebiotics, including fermented milk products that, apart from probiotic bacteria, contain numerous elements promoting their growth (e.g., lactose, oligosaccharides, α-lactoalbumin, lactoperoxidase, lysozyme and lactoferrin) [2,3,4]. Lactoferrin (LF, LTF) is a pleiotropic glycoprotein that is universally present in the organisms of mammals. LF is produced by the epithelial cells of mucosa and neutrophils and may be released into the circulation and surrounding tissues. LF, besides offering a wide spectrum of antimicrobial properties, regulates the function of the immune system’s bridging mechanisms of innate and acquired immunity. To date, via numerous investigations, 20 various physiological roles of LF have been confirmed [5,6,7,8,9,10,11]. One of these is a prebiotic action on beneficial bacteria in the gastrointestinal and genital tract [2,12]. 

This article describes the results of laboratory tests, animal studies and clinical trials, confirming the antimicrobial and prebiotic action of LF in the female genital tract. LF, through normalization of the genital tract ecosystem, allows infection and inflammation to be overcome and protects against complications in pregnancy, such as abortion or premature delivery.

## 2. Immunity of the Female Genital Tract

The internal wall of the vaginal canal is composed of stratified squamous epithelium, and the lamina propria of the mucosa is populated by immune cells, such as granulocytes, macrophages, dendritic cells (DC), natural killer (NK) cells, and B and T lymphocytes, which may form large conglomerates called lymphatic follicles (Figure 1B). Similar to other mucous membranes, both nonspecific and specific immune mechanisms act in the genital tract. NK cells protecting the genital tract against viral infection constitute 10–30% (up to 70% upon fertilization) of the local leukocyte population. Epithelial cells of the uterus and vagina bear Toll-like receptors (TLRs) that, following the binding of microbial antigens, deliver signals (among other things, to T CD4^+^ lymphocytes) stimulating them to produce cytokines and proteins of antimicrobial activity. Among these mediators are cathelicidin, calprotectin, elafin, lysozyme, surfactant protein A (SP-A), secretory leukoprotease inhibitor (SLPI) and LF; however, a key role is attributed to α- and β-defensins. Collectively, these proteins are described as host defense proteins and peptides (HDPPs). Many of them act synergistically for the enhancement of antimicrobial defense of the genital tract, for example, SLPI and lysozyme or LF and lysozyme [13,14,15,16]. Some of these proteins also exhibit anti-inflammatory activity, e.g., elafin and SLPI, which inhibit neutrophil and bacterial proteases and protect tissues against damage during the ongoing inflammatory process. When the mucosa is exposed to pathogenic microorganisms, the levels of HDPPs increase due to the activation of genes encoding these mediators, the proteolytic cleavage of inactive pro-peptides, or their supply by infiltrating neutrophils [17]. 

The activities of HDPPs depend on a particular phase of the menstrual cycle; thus, they are hormonally regulated [14,15,16,17,18]. For example, in the late proliferative (follicular) phase and the menstruation phase, an accumulation of neutrophils and high concentrations of elafin and SLPI are observed, which protects the endometrium against infection and favors its restoration. The production of defensins is also stimulated by estrogens and suppressed by progesterone. Likewise, the concentration of LF in the genital tract changes depending on the estrogen level, attaining the highest level in the late proliferative and early ovulatory phases and the lowest in the middle secretory (luteal) phase, when the estrogen concentration is lowest. DC are particularly active in the postmenstrual phase at a high estrogen level when the epithelium is thin and is most susceptible to infection. The macrophages, on the other hand, exhibit the highest activity before menstruation and after the penetration of sperm into the genital tract. TLR expression on the epithelium also depends on the menstruation cycle. The levels of TLR2, TLR4 and TLR9 increase before menstruation and, during the secretory phase, at a high progesterone level, the presence of TLR6 and TLR10 is upregulated. TLR2 is activated by the pathogenic *Neisseria gonorrhoeae*, and TLR2 and TLR4, by *Chlamydia trachomatis*. In addition, the secretion of immunoglobulins (Ig), abundantly residing in the lamina propria of the mucosa, is regulated by estrogen and progesterone [18]. In contrast to the gastrointestinal tract, which is dominated by secretory IgA (sIgA), in the genital tract, IgG prevails, although sIgA also occurs. 

## 3. Lactoferrin in the Female Genital Tract

Among HDPPs, LF possesses the widest range of antimicrobial and immunoregulatory properties. This pleiotropic protein from the transferrin family inhibits viral, bacterial, fungal, and parasitic infections. It acts, among other things, by binding and limiting the availability of iron essential for pathogen growth, damaging their cellular structures (cell wall and membrane), inhibiting the adhesion and binding of pathogens to the host’s cells, inducing apoptosis of infected cells, degrading virulent factors as a serine protease (for example, receptors and enzymes produced by pathogens), inhibiting biofilm formation by pathogenic fungi and bacteria on the epithelial surface, and forming reactive oxygen species (ROS), which are toxic for microorganisms [9,19,20,21]. In the latter case, the multidirectional action of LF is particularly evident. The protein has the ability to strongly bind Fe^3+^ ions and release them only in a strongly acidic environment (pH < 3.5). By sequestering iron, it deprives pathogenic microorganisms of the iron that is essential for their metabolic processes. On the other hand, under acidified environmental conditions during infection and inflammation, it releases iron ions that are necessary to catalyze the formation of ROS, resulting in killing pathogenic bacteria [22]. These direct ways of antimicrobial activity may be supported by indirect activity via the regulation of the immune response to infection. Such actions include enhancement of the immune response at its early phase (activation of inflammation to efficiently remove a pathogen) or its diminishment at a late phase (inhibition of inflammation results in tissue damage limitation and the acceleration of its regeneration) [9,10,23,24]. 

The regulatory action of LF on the immune system encompasses both innate and acquired immune processes, such as the maturation of T and B cells, Ig production, the regulation of activity of K and NK cells, granulocytes, monocytes, macrophages, DC and other types of immune cells, as well as the production of cytokines, chemokines, growth factors, metalloproteinases, ROS and other inflammatory mediators. LF may also accelerate the repair processes of damaged tissues and wounds [5,6,7,8]. Significantly, LF-derived peptides that are generated upon enzymatic degradation, for example, lactoferricin, also demonstrate similar or even higher activity than the native protein [25,26]. This is relevant when taking into account the partial digestion of LF in the gastrointestinal tract and other tissues, during ongoing inflammation or infection.

**Figure 2:**
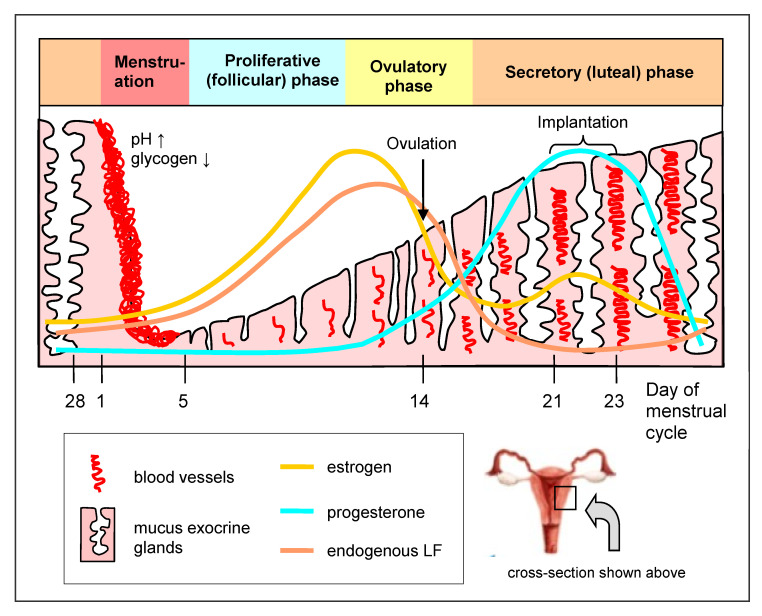
The changes in the uterine mucus (endometrium) during the menstrual cycle. In the proliferative (follicular) phase, the production of estrogen increases, but it decreases in the secretory (luteal) phase. The production of progesterone is high in the secretory phase and low in the proliferative phase. The production of LF is positively correlated with the estrogen level and negatively with the progesterone level. During menstruation, pH in the genital tract increases to 7.3–7.4, but glycogen production by epithelial cells is low, so the population of lactobacilli in the vagina and uterine cervix is low. Under such conditions, the genital tract is particularly prone to infection.

LF (as other antimicrobial proteins, mucins, and glycogen) is secreted by epithelial cells lining the uterus (corpus and cervix) and vagina into the cervicovaginal mucus, to facilitate uterine lubrication and microbial clearance (Figure 1B). In normal-cycling women, the LF content in the cervicovaginal fluid fluctuates with the level of circulating estrogen, which is increased during the proliferative (follicular) phase and decreased during the secretory (luteal) phase (Figure 2). LF gene expression in the genital tract is therefore induced by estrogen [18,27]. The epithelial cells lining the genital tract are not the sole source of LF; recruited circulatory blood neutrophils, which are attracted by local infection to remove infectious agents, also serve as a source. Neutrophils contain LF in the secondary granules and release it after activation into circulation and infiltrating tissues. Briefly, 10^6^ of the activated neutrophils release about 15 µg of LF [17,18]. In healthy women, low concentrations of LF (around 0.16–0.2 μg/mL) were found in vaginal and cervical secretions (cervicovaginal fluid) [28]. Another analysis showed higher and variable concentrations of cervicovaginal LF (8–154 μg/mL), depending on the phase of the monthly cycle [29]. 

LF is also present during pregnancy in amniotic fluid and the thick cervical mucus plug [30,31]. Amniotic fluid LF concentrations are quite low (1–2 μg/mL) early in pregnancy and rise markedly (to 5–15 μg/mL) from 32 weeks of pregnancy until term delivery. The levels of LF in the decidua and LF production probably rise as protective events against the inevitable contact with pathogens during normal labor [31]. A much higher (10–1000 µg/mL) LF concentration is found in the cervical mucus plug, which closes the uterine cervix and protects the uterus against contact with a non-sterile external environment, having particular significance during pregnancy [30]. The cervical mucus plug also contains a high concentration of SLPI and lysozyme (0.6–1 mg/g of the mucus) [30,32]. During pregnancy, the sources of LF in the genital tract are not only represented by secretory epithelial cells and neutrophils from the circulation but probably also by fetal tissues [17]. 

LF concentration in the cervicovaginal fluid increases during genital tract infection, which correlates with the number of tissue-infiltrating neutrophils [28]. An increased concentration of LF in the reproductive tract was observed, among others, during *N. gonorrhoeae*, *C. trachomatis*, *Trichomonas vaginalis*, and anaerobic bacterial infections and cervicitis. At the same time, in the cervicovaginal fluid, significantly higher levels of pro-inflammatory interleukin (IL)-1β were detected [33]. Significantly increased production of LF, IL-6, IL-1α, interferon (IFN)-α and IFN-β was detected in the cervicovaginal fluid of *C. trachomatis*-positive women. In infected patients, LF levels increased by about 5–6-fold (to 4.5 μg/mL from the 0.7 μg/mL found in healthy individuals). No association was observed between the levels of LF and cytokines and genital symptoms (increases were noted both in symptomatic and asymptomatic patients) [34]. In addition, the amniotic fluid LF levels were markedly elevated (to 2.7–10.4 μg/mL) in pregnant women prior to 32-week gestation with intra-amniotic infection [31]. A simultaneous increase in LF concentrations (from 0.86 to 8.96 μg/mL) and IL-6 in amniotic fluid was observed in pregnant women with chorioamnionitis (CAM). In addition, the immunohistochemical localization of LF in fetal membranes (amniotic and chorionic) was positively associated with leukocyte migration [35,36]. Salivary LF levels in neonates, delivered by mothers with CAM, were significantly higher than in mothers without CAM (3.4 vs. 0.8 μg/mL). The observation suggests that salivary LF may be one of the fetal defense factors against intrauterine infections [37]. Increased LF levels, determined in the cervicovaginal fluid, together with many other antimicrobial peptides, cytokines and chemokines, may be a valuable diagnostic marker of inflammation that could be useful in the diagnosis of female genital tract infections [38,39]. 

## 4. Normal Microbiota of the Female Genital Tract and Factors That Can Affect It

The nonspecific and specific immune mechanisms cooperate closely with bacteria that physiologically colonize the genital tract to protect against infection. Therefore, these microorganisms comprise an essential constituent of the environment of the genital tract in healthy women. The upper part of the female genital system (fundus and corpus uteri, oviducts and ovaries) is sterile and, in healthy conditions, does not contain any microorganisms since the cervix constitutes a physiologic barrier between the sterile and nonsterile environments of the genital system. Instead, the lower part of the female genital system (genital tract) is colonized by a set of microorganisms (described as microbiota of the genital tract) that populate a common ecological niche and remain in mutual associations with themselves and a host. These relations can be defined as a mutual symbiosis (advantageous for both partners), commensalism (benefit for one partner and no harm for the other), or parasitism (benefit for one partner and harm for the other). Members of the microbiota may communicate with each other by means of secreted mediators, in order to more effectively colonize the occupied niche (quorum sensing), for example, to form a biofilm resistant to antimicrobial agents. They may also react to the defense factors of a host by producing neutralizing substances, such as proteolytic enzymes degrading the LF molecule. In addition, they respond to other host mediators, among other things, adrenalin and noradrenalin, by producing factors of virulence [40]. 

The human organism is populated by about 40 billion (4 × 10^13^) bacterial cells, and a majority (99%) is contained in the intestinal tract [41]. In the genital tract, mainly in the vagina, their numbers are estimated at 10^2^–10^11^ cells in 1 mL of vulvovaginal fluid, with an over twofold prevalence of anaerobic over aerobic bacteria (5:2) [42]. Microbiota of the genital tract affects not only the health of a woman but also of her partner and child. Under healthy conditions, the microbiota is dominated by anaerobic Gram+ bacilli of the *Lactobacillus* genus (in particular, *L. crispatus*, *L. jensenii*, *L. gasseri*, *L. iners*) that constitute up to 95% of the whole vaginal ecosystem and protect against invasion by pathogenic microorganisms and subsequent infection. A meta-analysis of the studies, performed in 2014, univocally demonstrated that the microbiota dominated by *Lactobacilli* is associated with a lower probability for bacterial vaginosis to occur [43]. In most women, one or two species of the *Lactobacillus* genus predominate [18]. The prevalence of particular species is related to ethnicity and place of residence [15,44]. In the population of Polish women, a predomination of *L. acidophilus*, *L. fermentum*, *L. plantarum*, *L. delbrueckii*, *L. rhamnosus*, *L. gasseri* and *L. casei* is observed [44]. Among US residents, the vaginal ecosystem is dominated by *L. crispatus* and *L. jensenii*, and in Mexico, Canada, Japan, Turkey, Belgium and Sweden, by *L. crispatus* [42]. Apart from various strains from the *Lactobacillus* genus, the strains of *Streptococcus*, *Staphylococcus*, *Enterococcus*, *Bacteroides*, *Corynebacterium*, *Peptostreptococcus* species and others are found in this ecosystem. *Candida* and *Saccharomyces* yeasts (mainly *C. albicans*) may also be present, forming the vaginal mycobiota. When these microorganisms are present in the genital tract in small proportions, they are commensals and are therefore not harmful to health. Based on molecular techniques, the vaginal microbiota of healthy North American women (who represented four ethnic groups) was studied, leading to the identification of 5 types of unique microbial community state (CST): CST-I (dominated by *L. crispatus*), CST-II (*L. gasseri*), CST-III (*L. iners*), CST-V (*L. jensenii*) and CST-IV (characterized by low *Lactobacillus* prevalence and anaerobes from *Prevotella*, *Gardnerella*, *Atopobium*, *Sneathia*, *Mobiluncus*, *Megasphera*, *Corynebacterium* and *Streptococcus* species [45].

The physiological microbiota inhibits the colonization and growth of pathogens in different ways. It produces antimicrobial factors (for example, hydrogen superoxide and bacteriocins) that directly damage and destroy undesirable anaerobic bacteria. The vaginal ecosystem is, to a large extent, regulated by sexual hormones. In the vaginal epithelium, estrogens induce synthesis of a high amount of glycogen, metabolized by *L. acidophilus* bacilli to produce lactic acid, which lowers the pH to 3.5–4.5, and in turn, inhibits colonization by pathogenic microorganisms. Lactic acid may also kill *N. gonorrhoeae* and destroy the herpes simplex virus type 2 (HSV-2) and the human immunodeficiency virus (HIV) [46,47]. *Lactobacillus* cells have a capability to strongly adhere to epithelial cells and the mucosa that hinders the access of pathogenic bacteria to the tissue. At the same time, by occupying the living niche, they restrict the accessibility of nutrients for competing pathogenic microorganisms. Lactic acid also regulates the activity of the host immune system, e.g., by increasing the production of the anti-inflammatory IL-1 receptor antagonist (IL-1RA) by the vaginal epithelium and by the inhibition of pro-inflammatory factors, such as IL-6, IL-8, tumor necrosis factor (TNF)-α, regulated on activation, normal T cells expressed and secreted (RANTES), and macrophage inflammatory protein-3 α (MIP3α), or by activating T helper 17 (Th17) lymphocytes after exposure to bacterial lipopolysaccharide (LPS) [15]. 

The genital tract microbiota is not constant with regard to quantity and quality and may change during a woman’s life under the influence of numerous, short- and long-term endogenous and exogenous factors. Basically, the microbiota changes during the lifetime. Immediately after delivery, under the influence of maternal estrogens, the infant’s microbiota resembles the woman’s microbiota in the reproductive term. Later, during prepuberty, the genital tract is characterized by a more alkaline condition (pH 7–8), a scarcity of *Lactobacillus* spp. and a predominance of *Bacteroides*, *Peptococcus*, *Porphyromonas*, *Diphteroids* species, *Staphylococcus epidermidis*, and the *Gardnerella vaginalis* genus [42]. Similar changes, associated with an estrogen deficit, are associated with women in the menopausal and postmenopausal terms. The changes distinctly correlate with strenuous symptoms in this period, such as the vaginal dryness associated with the inflammation, thinning and disturbance of the integrity of the vaginal epithelium [48]. During menstruation, the numbers of *Lactobacillus* cells transiently decrease and the pH of the vagina increases to 7.3–7.4 [42]. The microbiota composition also alters during pregnancy due to hormonal changes. In pregnant women, the domination of *L. iners*, *L. jensenii*, *L. johnsonii* and *L. crispatus* has been confirmed. The microbiota is also subject to changes during the advancements of pregnancy and depends on age [18]. In general, the microbiota becomes more stable and less differentiated with regard to the variety of species, with a distinct prevalence of *Lactobacillus* [44]. 

Hormonal contraception essentially affects both the microbiota and genital tract immunity. The direction of these changes depends on applied therapeutics. In a large, retrospective study on fertile women (*n* = 682) on progestin-based therapy, a domination of undesirable bacteria, predisposing to a disruptive balance in vaginal microbiota (dysbiosis), was demonstrated. In turn, women applying a combination of estrogen and progestin had a higher proportion of *Lactobacillus* spp., generating hydrogen superoxide (*L. gasseri*, *L. crispatus*, *L. jensenii*), which ensures a healthy balance in the vaginal microbiota (eubiosis) [18]. Hormonal contraception also changes the immune response of the genital tract, although the results of studies are divergent since both inhibition, as well as stimulation of the proinflammatory cytokine secretion, were found. It was also demonstrated that changes in the inflammatory response in the genital tract are associated with a higher risk of infection, e.g., HIV and *Candida* spp. [18].

Other factors changing the microbiota of the genital tract are listed below:

- Antibiotic therapy (antibiotics killing Gram-positive bacteria, among others penicillin, cephalosporin, carbapenem, macrolides, polimyxin, bacitracin, vancomycin);
- Natural corticosteroids (secreted upon stress) and steroidal drugs;
- Nonsteroidal anti-inflammatory drugs (NSAIDs);
- Immunosuppressive drugs, radiotherapy;
- Chronic inflammatory illnesses including those of autoimmune etiology, and metabolic syndrome (diabetes, obesity, hypertension);
- Diseases with primary or secondary immunosuppression (for example, oncologic diseases);
- Infectious diseases (viral, bacterial, fungal, parasitic);
- Surgeries of the urinogenital tract;
- Mechanical contraception (rings, uterine spiral);
- Sexual life (in particular early-initiated, frequent, with various partners, oral and anal sex and digital vaginal penetration);
- Pregnancy;
- Hygiene habits (scant or excessive hygiene, application of tampons, disinfecting agents, vaginal irrigation);
- Lingerie (tight-fitting, cool, synthetic);
- Low socioeconomic status;
- Cigarettes, alcohol, narcotics;
- Diet (mainly monosaccharides);
- Microbiota in the oral cavity;
- Bathing in pools.

## 5. Abnormal Microbiota of the Female Genital Tract

As mentioned above, changes in the vaginal microbiota composition lead to dysbiosis, where protective lactobacilli are dominated by anaerobic bacteria or fungi (commensal, opportunistic, or pathogenic) (Figure 1C). The risk of vulvovaginal infections (VVI) substantially increases when the number of *Lactobacillus* spp. cells declines below 10^6^ CFU/mL of the vaginal discharge. Such a condition leads to dysbiosis and, consequently, depending on the prevailing group of pathogens, to the development of [15]:

- Bacterial infection without inflammatory state (bacterial vaginosis, BV);
- Bacterial infection with the domination of aerobic bacteria and accompanying inflammatory state (aerobic vaginitis, AV);
- Fungal inflammation of the vulva and vagina (vulvovaginal candidiasis, VVC);
- Trichomoniasis of the vagina (*Trichomonas* vaginitis, TV);
- Non-typical inflammation (*C. trachomatis*, *Mycoplasma hominis*, *Mycoplasma genitalium*, *Ureaplasma urealyticum*, and others).

Dysbiosis often leads to other types of dysbioses since, often, mixed inflammations are found (a concurrent occurrence of AV and TV, AV and BV or AV and VVC) and foster infections (e.g., *T. vaginalis*, *C. trachomatis* and *N. gonorrhoeae*). Infections of the genital tract most frequently occur in women, with more than half of the world’s population having experienced or who will experience at least one infection of this kind. What is more, these infections usually have a chronic or remissive character and represent a serious problem, not only from epidemiologic and clinical aspects but also from psychological and social ones [15]. Genital tract inflammation increases the danger of the occurrence of sexually transmitted infections (STIs), such as infections by human papillomavirus (HPV), herpes simplex virus (HSV), HIV, hepatitis B virus (HBV) and hepatitis C virus (HCV). In addition, inflammation increases the risk of pelvic inflammation, endometritis, infertility, extrauterine oviductal pregnancy and chorioamnionitis, premature rupture of the membrane (PROM), miscarriage and premature deliveries, with consequences for neonates and infections of neonates during delivery [44,49]. 

In AV, due to a decline in the numbers of *Lactobacillus* cells, an expansion of aerobic bacteria occurs with an increase in pH value to 5.5–6.5, as well as the development of vaginal mucous layer inflammation. In the course of AV, the most frequently found microorganisms belong to *Streptococcus agalactiae* (group B *Streptococcus*) and *Escherichia coli*, as well as *Staphylococcus* and *Enterococcus* species. These microbiotas may derive from the gastrointestinal tract, due to the proximity of the openings of both systems. Some authors are of the opinion that AV results from primary immune disturbances of the genital tract or that this is a dermatologic vaginal disease that is subsequently complicated by an incorrect overgrowth of vaginal microbiota [50]. In fact, 5–23% of women of reproductive age suffer from AV [51]. 

BV, in turn, usually results from the growth of opportunistic (relatively pathogenic) and pathogenic, mainly anaerobic bacteria from the genus *Gardnerella vaginalis* and *Atopobium vaginae*, as well as *Anaerococcus*, *Bacteroides*, *Prevotella*, *Leptotricha*, or *Peptostreptococcus* species. These bacteria have the ability to establish firm adherence to the epithelium so that they frequently create a biofilm on the mucosa of the genital tract. This pathology is not accompanied by typical inflammation; hence, it is termed vaginosis and not vaginitis. The absence of inflammation is a consequence of leukocyte migration inhibition by lipid acids (chiefly, succinic acid) released by pathogenic bacteria [52,53]. A relatively high proportion of women suffers from confirmed BV; that is, 25–30% of women of reproductive age, according to the epidemiologic data from the European Union and the United States [49]. 

VVC, on the other hand, is even more common. About 75% of women, at least once in their lifetime, have experienced one VVC episode, and 40–45% have experienced two or more. VVC is caused by the proliferation of yeast cells and transition to an invasive hyphal form of *Candida* spp. (mainly *C. albicans*, more rarely, *C. krusei* and *C. glabrata*), and biofilm formation, often with a dominance of *Lactobacillus* at the same time. VVC may also be acquired by a fecal route [15,51]. 

Repeating episodes of BV and VVC facilitate infection with *C. trachomatis*. This intracellular pathogen is responsible for a large proportion of infections of the genital tracts in both sexes and, annually, about 45 million new infections are confirmed worldwide. In 80% of women, the infections remain without symptoms and are, hence, easily transferred by a sexual route to other individuals. Although *C. trachomatis* does not cause strenuous symptoms, it often leads to inflammation of the uterine cervix, oviducts and endometrium (endometritis); it may also infect newborns, causing conjunctivitis and lung inflammation [54].

Although routinely applied antibiotics (metronidazole, clindamycin, azithromycin, doxycycline) and antifungal drugs (clotrimazole, fluconazole) are effective in the short-term therapy of BV and VVC, respectively, their usage is restricted due to possible side-effects. They include the development of candidiasis or oxidative inflammation of the vagina caused by the destruction of the natural microbiota, and a particularly dangerous growth of antibiotic-resistant strains, as well as the relapse of so-called superinfection, which is resistant to classical therapy. Although in accordance with present recommendations, the treatment of VVC with fluconazole should be continued for 6 months, the relapses occur in as many as half of the cases. BV, when treated with metronidazole, led to relapses in 30% of patients after 3 months, and in 60% after 6 months, following cessation of the therapy [51].

The relapsing feature of these illnesses, despite proper diagnosis and directional therapy, inclines practitioners to a repetition of the therapeutic procedures that intensifies dysbiosis and leads to subsequent relapses (a vicious circle effect). Negligence in the implementation of such an important part of the therapy as quantitative and qualitative restoration of genital tract eubiosis may be a frequent cause of failures in antibiotic therapy. Such a therapeutic procedure should, therefore, be essential in the prophylaxis and therapy of VVI. The physiologic balance of the genital tract microenvironment may be restored by the application of probiotics and/or prebiotics, and optimally by both these elements (synbiotics). 

## 6. Probiotics and Prebiotics, Activity and Safety

Probiotics, according to the definition of the World Health Organization (WHO), are “live microorganisms which when administered in adequate amounts confer a health benefit on the host” [55]. Usually, as probiotic strains, bacteria from *Lactobacillus* and *Bifidobacterium* species and fungi from the nonpathogenic *Saccharomyces* yeast species, are used. Their wholesome actions have been demonstrated in numerous studies. These actions include the regulation of the gut microbiota, resulting in the protection and combat of gastrointestinal infections, post-antibiotic diarrhea, symptoms of irritable colon and nonspecific inflammatory gut diseases, and allergy (e.g., atopic skin inflammation). In addition, probiotics may also regulate the function of the immune system, as well as lowering cholesterol and blood pressure levels. 

Many of these activities may be ascribed to the metabolic products of probiotic bacteria. They include vitamins from the B group, vitamin K, organic acids (acetic, butyric, propionic), exhibiting anti-inflammatory and trophic actions for colonocytes, gamma-aminobutyric acid (GABA), a main neurotransmitter in the central nervous system, generating in the fermentation process bioactive peptides (antimicrobial, immunomodulatory, hypotensive, anti-coagulant and anti-oxidant), bacteriocins (peptides of antibiotic action), enzymes (e.g., β-galactosidase digesting lactose), anti-inflammatory, antioxidant, anticancer and antiteratogenic conjugated linoleic acid (CLA), as well as bifidogenic exopolysaccharides [4,56,57]. When ingested orally or used vaginally, probiotics are generally considered safe and well-tolerated. However, their efficacy and safety characteristics are strain-specific, so those characteristics demonstrated in a clinical study for a given probiotic strain cannot be extended to others. Probiotics have been recognized as safe by the Food and Drug Administration (FDA) and European Food Safety Authority (EFSA). They may be applied in conventional food, specially designed healthy diets (i.e., with the addition of bioactive ingredients), or as dietary supplements, as well as specially dedicated preparations or drugs. 

Prebiotics, on the other hand, are defined by the International Scientific Association for Probiotics and Prebiotics (ISAPP) as “non-viable substrates that serve as nutrients for beneficial microorganisms harbored by the host, including administered probiotic strains and indigenous (resident) microorganisms” [58]. These are usually plant-based products, such as polysaccharides (inulin, fructo- or galactooligosaccharides-FOS, GOS) or non-sugar compounds that are not digested by the host’s enzymes but are fermented by the microbiota, and improve the condition of the host. Dairy products (most commonly yogurt) may also be valuable sources of prebiotics in our diet. Prebiotic properties are exhibited by phosphates, lactose, oligosaccharides (mainly these containing N-acetylglucosamine), nucleotides, α-lactoalbumin, lactoperoxidase, lysozyme, glycomacropeptide (GMP) and LF [3,4,59].

## 7. Probiotics and Prebiotics for Prophylaxis and Therapy of Female Tract Infections

At present, probiotics and prebiotics are commonly used in gynecology. Although women, for centuries, have intuitionally enhanced their intimate health condition with fermented food, scientists only turned their attention to such diets as recently as 30 years ago. In one of the first clinical trials, patients with recurrent VVC took 250 g of yogurt containing *L. acidophilus* daily. Following 6 months of the treatment, significantly lower colonization of the vagina and anus by anascogenic yeasts was confirmed and the index of the disease recurrence fell from 2.54 to 0.38 of cases [60]. At this point, dozens of clinical trials have been conducted that, in the majority of cases, confirmed the efficacy of such treatment.

Trivagin^®^ preparation (containing probiotic strains most frequently found in Polish females: *L. rhamnosus*, *L. gasseri*, *L. fermentum*, *L. plantarum*), applied by an oral route, was effective in restoring and maintaining normal vaginal flora in Polish women (*n* = 60) with recurrent BV. Signs and symptoms of BV were resolved in 65% of the patients, and a subjective improvement was seen in 91% of the patients, according to the Amsel criteria [61]. In a randomized clinical study in Norway and Denmark, 100 women with bacterial vaginosis, diagnosed by the Amsel criteria, were treated with EcoVag^®^ vaginal gelatin capsules, containing two different strains of probiotic lactobacilli, for 3 months after vaginal clindamycin therapy. In total, 65% of the lactobacilli-treated women were still BV-free compared to 46% of the placebo-treated women at the end of the 6-month follow-up [62]. Florisia^®^ vaginal tablets (*L. brevis* CD2, *L. salivarius* FV2, *L. plantarum* FV9) were effective in the 7-day treatment of Italian women (*n* = 34) affected by symptomatic BV, in a randomized clinical trial. The treatment was effective in eliminating BV and in restoring normal vaginal flora in 61% and 50% of patients, respectively, compared with 19% and 6% of patients in the placebo group, as determined 2 weeks after cessation of the therapy [63]. The same preparation, applied in Indian BV-affected women, reduced proinflammatory cytokine levels in the cervicovaginal fluid. In an in vitro test, Florisia^®^ probiotic lactobacilli showed a bactericidal effect on opportunistic bacteria (*Enterococcus faecalis* and *S. epidermidis*) and prevented sperm lipid peroxidation induced by ROS and its detrimental effects on sperm function, such as motility and viability. Vaginal probiotics may, therefore, protect sperm against toxic ROS under conditions of inflammation of the female genital tract [64,65].

In systematic reviews, the efficacy of single or “cocktail” probiotic strains, administered orally or intravaginally, either alone or in conjunction with antibiotics for the treatment of BV, was analyzed [66,67]. The authors did not provide sufficient evidence for or against recommended probiotics for the treatment of BV, due to substantial heterogeneity in probiotic products, trial methodologies and outcome measures, and concluded that well-designed randomized controlled trials with standardized methodologies and larger patient cohorts were needed. In addition, they claimed that the oral metronidazole/probiotic regimen and the probiotic/estriol preparation had beneficial outcomes for a microbiological cure [67]. The preferred route of delivery for probiotic lactobacilli is intravaginal. Probiotic preparations containing high doses of lactobacilli were effective in some clinical trials, suggesting that, besides strain characteristics, the amount of exogenously applied lactobacilli could have a role in their effectiveness [66]. A recent meta-analysis included 10 randomized controlled trials (RCTs) (*n* = 2321) that compared the efficacy of the treatments with probiotics versus placebos in BV patients. The therapies, applying only probiotics, had beneficial outcomes both in the clinical cure rate and Nugent score on the 30th day, and this effect lasted for 8 weeks. Probiotic-post-antibiotic therapy only had a beneficial effect in the short term [68].

Systematic reviews of several clinical studies of probiotic use in more than 1500 (11 RCTs) and more than 3500 (8 RCTs) pregnant and lactating women, respectively [69,70], indicated that exposure to probiotic strains during pregnancy was safe for both mother and fetus, without an increase in adverse outcomes such as miscarriages or malformation incidence, and without significant consequences for gestational age, cesarean section, and birth weight. Probiotics are rarely systemically absorbed and are not expected to be transferrable to breast milk. There are no published data regarding adverse events (AEs) in breastfed infants [69,70]. The dietary application of multistrain probiotics VSL#3 (a mixture of *Lactobacillus*, *Bifidobacterium* and *Streptococcus* strains) during late pregnancy improved some immune parameters and vaginal microbiota, suggesting a potential anti-inflammatory effect on vaginal immunity and possible implications in preventing preterm birth, as indicated in a pilot clinical study of healthy pregnant women (*n* = 27) [71].

Fewer clinical trials have investigated the efficacy of probiotics in the prophylaxis and treatment of VVC. In 2017, a systematic review analyzed the results of 10 randomized clinical trials (*n* = 1656) using probiotics as an adjuvant therapy for the antifungal treatment of VVC in non-pregnant women. According to the authors, probiotics as an adjuvant therapy could increase the short-term but not long-term clinical and mycological cure. Probiotics were safe and did not increase the incidence of serious or non-serious AEs. The researchers suggested a need for further RCT standard studies [72].

However, there have been no obligatory recommendations regulating the usage of probiotics in therapy related to the female genital tract. The most frequently applied gynecological probiotic strains belong to the *L. casei* subsp. *rhamnosus*, *L. reuteri*, *L. crispatus*, *L. plantarum*, and *L. gasseri* genera. They are isolated from the natural microbiota of healthy women, preferentially from the nationality/population where they will be applied. In other words, for example, for Polish patients, proper probiotics should contain strains isolated from Polish women. Their application increases the effectiveness of colonization of the genital tract and, consequently, the elimination of pathogenic microorganisms. Such preparations are available for Polish patients, with recommendations for prophylaxis and the assistance of therapy in infections of the urogenital tract [42]. Gynecological probiotics should contain a strictly defined bacterial strain of confirmed activity, exhibit an ability to adhere to the vaginal epithelium and an antagonistic action toward pathogens, and colonize the gastrointestinal tract [42,44,73]. Probiotics only temporarily colonize the epithelium of the genital tract, changing the composition of the local micro-environment to a milieu inconvenient for pathogen growth, one that promotes restoration of the correct microbiota and elimination of the infection [44,74,75]. Probiotic colonization is also hindered by sexual activity (vaginal intercourse and the presence of semen) [74]. Endogenous factors that hinder the colonization of probiotic strains are resident symbiotic strains and components of the local immune system that ultimately determine the quantitative and qualitative composition of the organism microbiota [44,76].

For improving their efficiency, it is worthwhile to enrich probiotics with other substances of prebiotic properties, including LF. Such complex probiotic-prebiotic preparations (synbiotic) may be applied in monotherapy or to assist treatment in conventional therapy by antibacterial or antifungal means. The preparations can be applied to the vulva/vagina/uterus (as gels, creams, ointments, tampons, sanitary towels, vaginal tablets, powders, or suppositories) or orally (as tablets, capsules, or powders) [77]. In both cases, effective temporary colonization of the applied strains in the vagina is observed. Local application is desirable during active, acute infections since it ensures the administration of a high number of bacteria directly to the destination, leading to their rapid colonization and combat against pathogens. An additional advantage of vaginal preparations derives from their supplementation with substances supporting the treatment of infections, by mitigating inflammation and reconstructing damaged vaginal epithelium (e.g., hyaluronic acid, ascorbic acid, or folic acid) [75]. Oral probiotics can also colonize the genital tract. Orally administered *Lactobacillus* strains have been recovered from the vagina [78]. Oral administration, in turn, provides an additional benefit in the form of gastrointestinal tract colonization and the prophylaxis of recurrent infections of the genital tract, since bacteria and fungi, colonizing the colon, represent the main reservoir of urogenital infections. The normalization of gut microbiota may, therefore, prevent recurrent gynecological infections [42]. What is more, oral application is not only more convenient for a patient but also generates other beneficial effects, such as a regulation of the immune response within the intestinal and genital tracts, that may have a long-term benefit in preventing the recurrence of infection [42,75].

## 8. Antimicrobial and Prebiotic Activity of Lactoferrin in the Genital Tract—In Vitro Tests

The beneficial action of LF on symbiotic resident bacteria, or when administered as probiotics, is multidirectional, and occurs, among other methods, by stimulation of their growth, facilitation of adhesion, and biofilm formation on the mucosal surfaces. At the same time, LF inhibits growth and activity or kills pathogenic microorganisms. Regulation of the immune system function is also of importance. The prebiotic activity of LF has been confirmed in several laboratory in vitro studies (cell culture models). The observed results depended on the type of LF used and the culture conditions, suggesting possible mechanisms of action of the protein.

**Figure 3:**
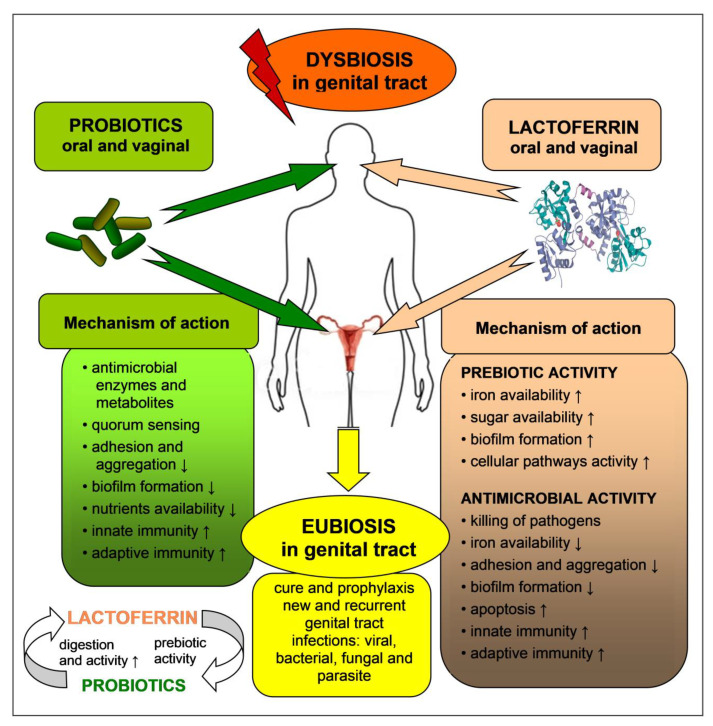
Joint activities of oral and vaginal probiotics and LF in the prophylaxis and cure of feminine genital tract infection and inflammation. Prebiotic and antimicrobial activities of LF are complementary. LF, via several means, destroys/inhibits the growth of pathogenic microorganisms and, at the same time, promotes the growth of symbiotic microorganisms, thus normalizing the composition of the vaginal microbiota. LF has beneficial effects on resident symbiotic bacteria and exogenous probiotic bacteria when applied orally and vaginally. LF is advantageous for the activity of probiotics. Probiotics, in turn, act enzymatically on LF by degrading the protein into more active peptides (lower left part), ↑ increase, ↓ decrease.

Iron-free bovine LF (apo-BLF) stimulated the growth of two strains of *L. rhamnosus* and *L. acidophilus* but inhibited the multiplication of the other tested strains of lactobacilli (*L. reuteri*, *L. rhamnosus*, *L. coryniformis*), *Bifidobacterium* (*B. bifidum*, *B. longum*, *B. lactis*, *B. infantis*), and the pathogenic bacteria (*E. coli*, *Staphylococcus aureus* and *Salmonella enterica)*. However, the minimal inhibitory concentrations (MIC) varied considerably; for the probiotic bacteria, it was ≥128 mg/mL, and for the pathogens, 4–32 mg/mL [79]. Similar observations were derived from tests using 10–20% iron-saturated BLF. The protein, added to the cultures at concentrations of 0.6–40 mg/mL, significantly inhibited the growth of the enteric pathogens, *Listeria monocytogenes*, *S. aureus*, *S. enterica* ser. Typhimurium and *E. coli*, without altering the proliferation of the symbiotic bacteria, *L. acidophilus*, *L. plantarum*, *L. reuteri*, *L. rhamnosus*, *B. lactis* and *Pediococcus acidilactici*. A synergistic inhibitory effect of LF and *L. reuteri* culture supernatants was also observed [80]. These observations suggest that iron-free and partially iron-free LF obliquely capture small amounts of iron available in the culture and, thus, limit the proliferation of the pathogenic bacteria. On the other hand, LF does not alter or stimulate the growth of lactobacilli, which are not absolutely iron-dependent, and may replace iron with manganese ions (which have analogous functions as iron in terms of being cofactors for the activity of numerous cellular enzymes) [81]. The ability to inhibit pathogen growth, without affecting probiotic bacteria, was also demonstrated by peptide fragments of the LF molecule. Pepsin hydrolysates of LF inhibited the proliferation of *E. coli*, *S. enterica* ser. Typhimurium, *E. faecalis*, *S. aureus*, but not the probiotic strains (*L. rhamnosus*, *L. reuteri*, *L. fermentum*, *L. coryniformis*, *L. acidophilus*, *B. infantis*, *B. bifidum* and *P. acidilactici*). Importantly, LF hydrolysates and supernatants from cultures of probiotic bacteria acted synergistically [82]. It is possible that LF hydrolysates enhanced the action of the antimicrobial compounds secreted by the probiotic strains. LF hydrolysates may be formed not only by the action of endogenous digestive enzymes in the stomach and intestine but also by the proteolytic activity of symbiotic/probiotic bacteria residing in these organs. Thus, probiotics and LF mutually enhance their effects (Figure 3). It was found that LF molecules were hydrolyzed by peptidases of, e.g., *Streptococcus thermophilus* and the *Lactobacillus delbrueckii* subsp. *Bulgaricus* strains that are used for milk fermentation in the dairy industry [83]. 

In a Korean study, a mixture of *L. acidophilus* GLA-14, *L. rhamnosus* HN001 and milk BLF in the probiotic mixture Respecta^®^ (Nutribioscence, Seoul, Korea) inhibited the growth of pathogenic *G. vaginalis* and *A. vaginae*. In a similar model, inhibition of the adhesion of *G. vaginalis* to HeLa cells was observed [84].

In a subsequent investigation, Italian researchers determined the effect of exogenous BLF on the interaction of *L. acidophilus* GLA-14 and *L. rhamnosus* HN001 with human epithelial cells derived from the uterine cervix (tumor HeLa cell line) [85]. BLF, at a concentration range of 0.5–4 mg/mL, was used in the study. Although no effect of LF on bacterial growth was determined, the protein augmented the ability of bacteria to form a biofilm, both on abiotic hydrophobic surfaces (polystyrene) and hydrophilic glass, as well as on the monolayer of HeLa cells. In the presence of BLF, the bacteria preferably adhered to the studied surfaces and they also formed a more extracellular matrix, enabling an aggregate formation that arranged compact, special, multilayer structures of bacterial biofilm. The formation of biofilm by probiotic bacteria is a desirable feature since it helps them to colonize and survive on the host’s epithelium and protect against population by pathogenic bacteria. Probiotic bacteria constituting biofilms are also resistant to the killing action of antibiotics. The ability of adherence and formation of a compact biofilm on the vagino-uteral epithelium, as well as their antibacterial activity toward those pathogens responsible for vaginitis, belong to the basic parameters tested in vitro during the evaluation of *Lactobacillus* strains as potential intravaginal probiotics [73].

In addition, in the studies of other researchers, the protective action of BLF with *L. brevis* and *L. crispatus* in an infection of cells with the intracellular *C. trachomatis* bacteria was confirmed [86]. Both *Lactobacillus* strains, when added to a HeLa cell culture, suppressed the adhesion of invasive forms of *C. trachomatis* (i.e., elemental bodies) and their penetration into the host’s cells. The strongest inhibition occurred when BLF (100 µg/mL) was present in the culture, although BLF alone did not affect the pathogen adhesion. In addition, both *Lactobacillus* strains significantly restricted the intracellular multiplication of *C. trachomatis*, but LF did not exhibit an additive action in this process. However, the monitoring of the whole infectious cycle of *C. trachomatis* revealed a strong inhibition of this process by separate *Lactobacillus* strains and an additive effect of LF. What is more, immunomodulatory properties of *Lactobacillus* and LF were also found. Both lactobacilli strains, as well as LF, significantly suppressed the production of proinflammatory cytokines, such as IL-6 and IL-8, by infected epithelial cells, and LF also exhibited an additive action in this process. Both cytokines are strong inducers of inflammation by activating neutrophils and acute-phase proteins. Although an acute inflammatory state is expected and is beneficial for the efficient elimination of infectious agents, in the case of an infection by *C. trachomatis*, a long-lasting infection often develops, accompanied by the constant excessive production of inflammatory mediators and tissue damage, which leads to serious complications including adhesions, dysplasia, infertility and chronic abdominal pain. As the authors conclude, a combinatory application of the prebiotic strains of *Lactobacillus* and LF in the treatment of genital tract chlamydiosis may not only effectively combat the infection but also protect women against the considerable complications caused by chronic inflammation [86]. Human LF (HLF) added at physiological concentrations (0.1 or 1 μg/mL) to primary cultures of human amniotic cells inhibited LPS-induced IL-6 secretion; therefore, one can speculate that endogenous amniotic LF can function as a local anti-inflammatory agent during chorionic and amniotic infections [35,36]. 

Liao et al. studied the antifungal activity of BLF-producing probiotic strain *L. casei*/pPG12.1-BLF, harboring the BLF coding sequence, and secreted the protein into agar. In a two-layer agar dish assay, which allowed *C. candida* to be directly exposed to a secretion of the modified *L. casei* strain, the number and average size of *C. albicans* colonies were lower than in the presence of the non-modified *L. casei* strain, so LF and lactobacilli acted synergistically [87]. 

Human and bovine LFs and LF-derived peptides also have the ability to stimulate the growth of various species of *Bifidobacterium*, symbiotic gut bacteria, as confirmed in several subsequent in vitro tests [88,89,90,91,92]. Although the molecular mechanism of the prebiotic activity of LF and peptides is not fully understood, it may involve the utilization by symbiotic/probiotic bacteria of (i) iron and (ii) sugar molecules (oligosaccharides) released from the LF molecule, (iii) the binding of LF to bacterial cell surfaces and, thereby, the activation of cellular signaling pathways and transcription factors, and (iv) the stimulation of adhesion and biofilm structure formation on epithelial surfaces [2,4].

As already mentioned, the restoration of vaginal ecological equilibrium is influenced not only by the prebiotic effect of LF but also by its ability to inhibit the growth of and kill the pathogenic resident microorganisms. Human and bovine LF and related peptides can inhibit the growth and kill those cells of *C. albicans* responsible for VVC, with an effectiveness similar to that of standard antifungal drugs. The mechanism of action includes the reduction of iron ion availability, direct destruction of the cell membrane and wall, the inhibition of glucose uptake, DNA and protein synthesis by fungal cells, and the induction of their apoptosis. LF acts synergistically with antifungal drugs [93,94,95,96,97]. LF and peptides also have bacteriostatic and bactericidal effects on those bacteria responsible for reproductive tract infections: *Staphylococcus* spp., *Streptococcus* spp., and *E. coli*. Synergistic effects with lysozyme and antibiotics have also been observed. The mechanism of such activity includes: (i) the sequestration of iron ions, (ii) cell wall and membrane damage, (iii) inhibition of adhesion to host cells, (iv) inhibition of biofilm formation, (v) inhibition of virulence factors formation and activity, (vi) induction of toxic ROS formation and (vii) apoptosis of infected cells. LF also increases the activity of immune cells that are responsible for fighting infections, including antigen-presenting cells, neutrophils, macrophages, NK cells, and lymphocytes [7,21,25,98]. The results obtained in in vitro tests were supported by clinical studies—LF was effective against these infections in both animals and humans [99,100,101,102,103,104]. 

## 9. Antimicrobial and Prebiotic Activity of Lactoferrin in the Genital Tract—In Vivo Tests

The investigation on mice with experimentally elicited BV (infection with *G. vaginalis*) showed that a combination of two prebiotic strains, *L. acidophilus* GLA-14 and *L. rhamnosus* HN001, with the addition of BLF (Respecta^®^), administered orally or intravaginally, ameliorated symptoms of the infection [84]. By means of quantitative PCR, the presence of the administered probiotics in the mouse vagina following both application routes was confirmed. In both models, a diminishment of the histological changes within the vaginal epithelium and a decline in the inflammatory state parameters, such as a lower expression of myeloperoxidase (MPO), inducible nitric oxide synthase (iNOS), cyclooxygenase 2 (COX2), NF-κB transcription factor and proinflammatory cytokines (IL-1β, IL-17, TNF-α), were registered. At the same time, there was an increase in the expression of IL-10, an anti-inflammatory mediator. The preparation also regulated the activity of T cells residing in the vaginal mucosa, by lowering RORγ expression (a transcriptional factor of proinflammatory Th17 cells) and increasing Foxp3 expression (a transcriptional factor of T regulatory cells (Tregs) that exhibit suppressor and repair functions. Importantly, the preparation was even more active upon oral administration, which suggests, according to the authors, that its main immunoregulatory action regarded innate and acquired immunity via the gut-associated lymphoid tissue (GALT), whereas its local effect in the vagina was lower [84].

In a VVC model of mice infected with *C. albicans*, the efficacy of recombinant BLF, produced by a probiotic strain of *L. casei*/pPG12.1-BLF, was also demonstrated [87]. In the prophylactic model, the mice were given the probiotic intravaginally and, after 2 days, were infected with *C. albicans*. A presence of BLF, synthesized by *L. casei*, was confirmed in the vulvovaginal fluid. The prophylactic application of the modified probiotic diminished the inflammation more significantly than the subsequent use of non-modified lactobacilli since, in the cultures, a fewer number of fungal CFU and filamentous pseudohyphae, adhering to the cornified epithelial layer in the vaginal smears, were found. In addition, a normalization of the immune response within the inflamed tissue was observed, because a drop in the level of the pro-inflammatory IL-17 and IL-23 in the cervicovaginal fluid was found. In addition, the number of CD4^+^ T cells in the draining lymph nodes declined, due to lower *C. albicans* cell numbers in the vaginal tissue. In the therapeutic protocol of this investigation, *L. casei*/pPG12.1-BLF was applied during a full-blown candidiasis infection. A similar efficacy of the restriction of infection and inflammation was found, evidenced by a drop of IL-17 and IL-23 levels in the cervicovaginal secretion and a decrease in CD4^+^ T cells in the draining lymph nodes. Overall, the efficacy of the modified *L. casei* strain was much higher than that of the wild strain and comparable to the therapeutic effect of clotrimazole. In conclusion, *L. casei*/pPG12.1-BLF was as effective as clotrimazole in treating VVC in a murine model [87]. 

The effectiveness of a similarly modified *L. casei* strain, bearing the gene of human LF and expressing recombinant human LF (rHLF), was demonstrated in a mouse model orally infected with a sublethal dose of *E. coli* [105]. rHLF/*L. casei* cells colonized the intestine, with a resulting restriction of pathogen numbers, weakened inflammatory states and histological changes in the intestinal tissue. The efficacy of the modified strain was ten times higher in comparison to the wild strain. In studies on newborn rats, the efficacy of a combined oral application of LF and the *L. casei* var. *rhamnosus* strain GG (LGG) in prophylaxis against *E. coli* infection was also confirmed [106]. The animals were given rHLF or LGG separately, or jointly, followed by oral infection with invasive *E.coli*. In the tissue and intestine exudates, more lactobacilli and significantly fewer pathogenic *E. coli* were found, and LF with LGG acted in synergy. Significantly, it was only in the intestine of animals from the rHLF/LGG group that maturing Peyer’s patches were identified. Thus, the applied preparation demonstrated not only antibacterial activity but also stimulated the development of the immune system of the immature suckling.

The efficacy of LF in the treatment of genital tract infections and their complications, for example, in preterm delivery, was demonstrated in several studies on mice and rabbits [107,108,109,110,111]. The inflammation and preterm delivery were induced by an intraperitoneal (i.p.) or intravaginal administration of LPS or *E. coli.* At the same time, the animals were given LF (BLF or rHLF) i.p. or intravaginally. In all infected or endotoxemic animals that were not treated with LF, preterm delivery occurred. Therefore, LF significantly prolonged the pregnancy by the inhibition of the inflammatory state in the genital tract, and in the whole organism. In parallel, a decrease in plasma and amniotic fluid IL-6 and TNF-α, as well as an improvement of histological parameters in the vaginal tissue, were determined. Lack of inflammatory exudates and necrosis in the endometrium, decidua and placenta, and cervical ripening was similar to that of healthy animals. The fetal survival was also better. In a subsequent study, the molecular mechanism of LF activity was explained. The protein, when applied i.p., suppressed LPS-induced endometritis in mice via the downregulation of the NF-κB pathway. The histopathological changes in the uterus, the activity of MPO, and the levels of nitric oxide (NO), TNF-α and IL-1β were also reduced [112].

In several clinical trials, including RCTs, a beneficial action of LF (mainly BLF) in women (*n* = 650) with dysbiosis of the genital tract and with confirmed bacterial and fungal infections that endanger preterm delivery, was demonstrated. The protein was administered orally and/or intravaginally, in prophylactic or therapeutic protocols, in monotherapy, or in combination with standard therapy for genital tract infections. In some of the trials, LF was applied, together with a probiotic preparation. In all studies, clinical and histological improvements were registered. The protein-optimized equilibrium of the vaginal ecosystem, due to the higher participation of symbiotic bacteria and a decline of pathogenic or relatively pathogenic microorganisms, ameliorated the inflammatory process and protected patients against threatening complications, such as PROM and preterm delivery [86,113,114,115,116,117,118,119,120,121,122,123,124,125,126,127]. The results of the clinical trials in women, involving the application of LF for vaginal infections and inflammation, are described in Table 1.

## 10. Lactoferrin in the Diet and Dietary Supplements

Endogenous LF acts beneficially on the symbiotic microbiota of all mucous membranes, including the urogenital tract. Additional LF may be supplied in diets with dairy products that contain the protein and its peptides. In Poland, in commercially available goat and bovine milk, the concentrations of LF do not exceed 0.1–0.3 mg/mL, which corresponds to 25–75 mg in a 200 mL cup. However, even at such low doses, the protein may be active by inducing myelopoiesis, regulating cytokine production [128,129], and diminishing postoperative shock in patients [130]. Hence, it is worthwhile to regularly include dairy products in the diet, in particular those manufactured from fermented milk (yogurt, kefir), taking into account the concurrent content of probiotic bacteria and assisting the growth of prebiotics (for example, lactose and LF). 

The quality of dairy products is evaluated by the content of bioactive ingredients, including LF. Most of them are prone to the thermal denaturation of proteins. Milk, subjected to standard pasteurization at 72–85 °C and microfiltration, preserves a majority (80–90%) of active LF. In contrast, in milk and its products sterilized at an ultra-high temperature (UHT treatment, involving heating up to 130–150 °C for several seconds at an elevated pressure), LF undergoes denaturation and loses its activity, including the ability to inhibit microorganism growth [131,132]. 

Numerous and proven wholesome properties of LF have inclined nutritionists to the inclusion of the protein as a dietary supplement and as an ingredient enriching functional food. In such a form, in many countries, LF has been commonly manufactured and distributed for more than four decades (in Poland, for about 15 years). In terms of the availability of the raw material, lactoferrin from bovine milk is produced since it shares a structure and properties with human LF [133]. BLF is isolated from milk by chromatography and is then lyophilized to obtain a pink powder, thanks to partial saturation (20–30%) with Fe^3+^ ions. At present, about 200 tons of BLF powder is produced worldwide [133].

The resistance of LF to digestion in the gastrointestinal tract is a separate problem. Although the protein, to a large extent, remains undigested in the gastrointestinal tract of newborns and infants, its fate in the gastrointestinal tract of adults has not been fully elucidated. The results of some studies suggest that LF is more resistant to digestion than other proteins, so only a small percentage of an orally administered dose may reach the intestine. In this location, LF and LF-derived peptides may act locally on the intestinal mucosa and residing immune cells, including Peyer’s patches, with lymphatic follicles being the main elements of GALT [134]. Activated lymphocytes relocate to the mucosal membranes of other organs, enhancing their immunity (integrated mucous resistance). LF may also activate various intestinal cells (both immunocompetent and not competent) for the production of immunologic mediators, such as cytokines, chemokines and neurotransmitters, that disperse to all organs and demonstrate the condition-systemic effects of orally administered LF [11,135,136]. Very recent results in vivo provided evidence that LF, when given orally to rats, exerts systemic effects [137]. After 3–24h following oral administration, changes in multiple gene expression were found, including IL-1 β, TNF-α, TNF-β, and leukocyte receptors such as CD40L, CD80, IL-5R. The changes were comparable with those induced by intravenously given LF. The results avert doubts regarding the systemic efficacy of LF when administered orally. 

Oral native LF or the products of its digestion (LF-derived peptides) can promote the growth of resident symbiotic bacteria, as well as of exogenous probiotic bacteria (applied in the diet or via dietary supplements) [2]. Oral probiotics can colonize the genital tract since orally administered *Lactobacillus* strains have been recovered from the vagina [78]. Thus, orally applied LF acts as a prebiotic on intestinal microbiota, and indirectly promotes beneficial vaginal microbiota. Oral LF application also generates other beneficial effects, such as a regulation of the immune response within the intestinal and genital tracts that may have a long-term benefit in preventing the recurrence of genital tract infection.

In the hitherto numerous in vitro and in vivo studies, no toxicity of LF and LF-derived peptides have been demonstrated. LF and its peptides, administered by various routes, e.g., orally, i.p., or intravaginally, did not evoke any undesirable responses. Bovine LF was classified by the FDA in 2000 as a product that is generally recognized as safe (GRAS) [138]. In 2012, the European Food Safety Authority (EFSA) also acknowledged BLF as a safe product [139]. Based on these documents, BLF may be used as a dietary supplement and ingredient of functional food. At this point in time, BLF is the only LF that is obtained on an industrial scale and is used as a functional food. Several rHLFs that have been produced in genetically modified organisms, such as bacteria, yeast, rice, potato, cows, etc., have not yet been approved for human consumption.

## 11. Conclusions

Probiotics, predominantly various *Lactobacillus* strains, are effective in the prophylaxis and therapy of dysbiosis and inflammation of the genital tract. Their efficacy may be improved by the concurrent application of prebiotics. These preparations act in a beneficial manner on the growth and activity of endogenous (resident) microbiota, as well as exogenous probiotic bacteria [18,65]. The prebiotic property is exhibited by lactoferrin—a multipotential glycoprotein, present in milk, the circulation and secretory fluids of mucosal membranes. Endogenous LF, released by the mucosa of the genital tract and resident/infiltrating neutrophils, protects this organ against invasion by pathogenic viruses, bacteria, fungi and protozoa. Its concentration in the vaginal fluid rises severalfold during inflammation and in such infections as cervicitis, bacterial vaginitis, gonorrhea, chlamydiosis and trichomoniasis, which confirms its protective role during these processes. The mechanism of LF action encompasses the inhibition of growth or killing of pathogenic microorganisms (antimicrobial activity) and the promotion of the growth and activity of symbiotic microorganisms (prebiotic activity) [7,12,18]. 

In addition, exogenously administered LF improves the condition of the genital tract by assisting in the equilibrium of the local (resident) microbiota (Figure 3). The efficacy of LF was confirmed in numerous tests in vitro, in animal models. and in human studies. LF, applied in the form of oral and intravaginal preparations, profitably altered the ecosystem of the genital tract by the elimination of pathogenic microorganisms and an increase in the population of *Lactobacillus* species that restored the state of eubiosis and protected patients against the dangerous consequences of dysbiosis, such as PROM, premature delivery, or miscarriage. LF may be applied with or without prebiotics, in monotherapy, or in the course of standard therapy with antibiotics and antifungal drugs. Such an approach is particularly effective in women with chronic and recurrent infections [12]. 

The application of LF is particularly recommended in such clinical situations when an increased susceptibility to genital tract infections occurs, e.g., during antibiotic therapy, anti-inflammatory treatment with steroids, long-lasting stress and infections, neoplastic diseases, radio- and chemotherapy, prior to gynecological surgery, and also in the peri- and postmenopausal period. In these cases, without any doubt, the prebiotic action of LF on the gastrointestinal tract microbiota plays a key role, which, consequently, improves the immunity of all mucous membranes [2]. Other, advantageous properties of the protein are also worth mentioning, such as the regulation of iron metabolism (including in pregnant women), protection of the gastrointestinal tract mucosa integrity, normalization of carbohydrate and lipid metabolism, promotion of bone formation and wound healing, as well as anticancer, analgesic, anti-stress and hypotension actions [7,23,140,141,142,143,144]. 

Bovine lactoferrin, isolated from milk, is commonly available as a dietary supplement and in functional food. The protein is safe and is well tolerated by patients. BLF has a GRAS status acknowledged by the FDA and EFSA [133]. For several decades, the protein has been commercially available worldwide, is easy to apply and relatively cheap, which additionally encourages its use in prophylaxis and therapy. Patients may take vaginal tablets with BLF only, or oral synbiotics with probiotics (mainly, several lactobacilli strains) and BLF. A large assortment of such preparations is commercially available on the international market. Gynecological synbiotics, composed of probiotic lactobacilli and prebiotic BLF, can also be purchased in some countries. They are available in the form of vaginal tablets. Numerous requirements must be considered during the development of probiotic products for the female urogenital tract so that they are considered safe [77]. 

## Figures and Tables

**Table 1:** Clinical studies with LF in the prophylaxis and therapy of women suffering from genital tract infection and inflammation.

Type of Study, Number of Participants, Country	Type of LF, Dose, Mode and Time of LF Application	Clinical/Laboratory Effects	References
Randomized, double-blind, placebo-controlled clinical study; *n* = 40 (women, healthy volunteers, of fertile age, 18–50 years); Italy	BLF RCX^TM^ (50 mg) plus probiotics (5 × 10^9^ CFU): *L. acidophilus* La-14 (ATCC SD5212) plus *L. rhamnosus* HN001 (AGAL NM07/09514) in Respecta^®^ complex (Giellepi S.p.A. Health Science, Seregno, Italy) in p.o. capsule Placebo: identical capsule containing 100 mg maltodextrin; 2 capsules/day, after breakfast, for 14 consecutive days	Vaginal swabs, collected at weeks 0, 1, 2 and 3 and analyzed for the consumed microorganisms by qPCR; vaginal pH determined Vaginal *L. acidophilus* and *L. rhamnosus* levels on days 14 and 21 ↑ Transient vaginal colonization (at least double levels from baseline) *L. acidophilus* on days 14 and 21, in 12 and 16, respectively, out of 20 women Transient colonization of *L. rhamnosus* on days 7 and 21, in 16 and 17, respectively, out of 20 women Vaginal pH (4.06–4.24) → No significant change in placebo group No AEs neither correlated nor unrelated	[113]
Randomized, double-blind, placebo-controlled clinical study; *n* = 40 (women of fertile age, 18–50 years, with symptomatic vaginal dysbiosis, signs or symptoms of vaginitis/vaginosis); Italy	BLF RCX^TM^ (50 mg) plus probiotics (5 × 10^9^ CFU): *L. acidophilus* GLA-14 (LMG S-29159) plus *L. rhamnosus* HN001 (AGAL NM07/09514) in Respecta^®^ complex (Giellepi S.p.A. Health Science, Lissone, Italy) in p.o. capsule Placebo: identical capsule containing 100 mg maltodextrin; 1 capsule/day, for 15 consecutive days	Vaginal swabs, collected at days 0 and 15 and analyzed for the consumed microorganisms by qPCR; symptoms, pH of vaginal secretions and Nugent score assessed through the study Symptoms (in particular, itching, vaginal discharge and fishy odor) ↓ Nugent score ↓ (from intermediate 5.0 to normal 2.9) Vaginal pH ↓ (from 4.42 to 4.09) Vaginal colonization of both probiotic strains at the end of the study ↑ No significant change in placebo group No AEs, neither correlated nor unrelated	[114]
Randomized, double-blind, placebo-controlled clinical study; *n* = 48 (adult women with recurrent BV, treated with metronidazole 500 mg twice daily for 7 days); Italy	BLF RCX^TM^ (50 mg) plus probiotics (5 × 10^9^ CFU): *L. acidophilus* GLA-14 (LMG S-29159) plus *L. rhamnosus* HN001 (ATCC SD5675) in Respecta^®^ complex (Giellepi S.p.A. Health Science, Lissone, Italy) in p.o. capsule Placebo: identical capsule containing 100 mg maltodextrin; 2 capsules/day, for 5 consecutive days, followed by a further 10 consecutive days at a dosage of 1 capsule/day (induction phase) During the maintenance phase (6 months following the induction phase), 1 capsule/day, for 10 consecutive days per month, starting the first day of menstrual cycle (prophylactic treatment) since the menstrual blood increases the vaginal pH and contributes to increasing the risk of BV recurrences	Normalization of Nugent score, remission of symptoms, recurrences during a 6-month follow-up period were assessed Nugent score ↓ Recurrence rate ↓ Symptoms of BV (vaginal discharge and itching) ↓	[115]
*n* = 6 (5 pregnant women and 1 non-pregnant woman, with a history of multiple pregnancy losses or preterm delivery and refractory BV *E. coli*, *Enterococcus*, *Gardnerella vaginalis*, *Staphylococcus*); Japan	BLF (NRL Pharma, Kawasaki, Japan) in intravaginal suppositories, 150 mg/day and p.o. tablets, 700 mg/day, starting before pregnancy or from 11th–21st gestational week until delivery	Normalization of vaginal flora (appearance and gradual predominance of *Lactobacillus*) Patients achieved pregnancy and delivered at term No AEs in mothers and newborns	[116]
Open, prospective, randomized clinical study; *n* = 60 (sexually active women aged 18–45 years, with symptomatic acute BV); Italy	BLF (AG Pharma s.r.l. Rome, Italy), in vaginal tablets 100 mg or 200 mg, 1 tablet/day for 10 days	Outcomes were a clinical evaluation based on Amsel criteria and Nugent scores Vaginal pH and structure of the vaginal bacterial biota and its dynamics during the study, determined by culture-dependent and molecular-based techniques Vaginal pH ↓ (2 weeks after stopping treatment, 60% and 89% of patients had pH < 4.5 in 100 mg and 200 mg of BLF, respectively) Nugent score ↓ (2 weeks after stopping treatment, 43% and 75% of patients had Nugent score ≤ 3 in 100 mg and 200 mg of BLF, respectively) Occurrence of vaginal bacteria species associated with BV: *Gardnerella*, *Prevotella* and *Lachnospira* ↓ Occurrence of *Streptococcus* spp., *Staphylococcus* spp., *Escherichia coli* ↓ Occurrence of *Candida* spp. ↓ Occurrence of *Lactobacillus* species ↑ Bacterial biota balance was maintained up to 2 weeks after treatment, was stopped only in 200 mg BLF-treated patients	[117]
Single-center retrospective cohort clinical study; *n* = 125 (pregnant women in the first trimester with BV diagnosis, with a history of prior spontaneous preterm birth); Italy	LF * in vaginal tablets, 300 mg/day for 21 days	Incidence of preterm birth < 37 weeks of gestation was the primary outcome Rate of preterm birth ↓ Gestational age at delivery ↑ (37.7 weeks in LF group vs. 35.9 weeks in control group without LF) Rate of admission for threatened preterm labor ↓ (45% vs. 70.8%) No differences in other outcomes (chorioamnionitis, preterm PROM < 34 weeks and neonatal outcomes) No cases of late miscarriage were reported No AEs were reported	[118]
Case report; 38-year-old multiparous women with 3 preterm PROM, diagnosed as having refractory vaginitis (*Streptococcus* group B and *Staphylococcus*), not cured with estriol and antibiotics; Japan	BLF (NRL Pharma, Kawasaki, Japan) in intravaginal tablets, 150 mg/day, and p.o. tablets, 700 mg/day, for 41 weeks (13 weeks before pregnancy and 38 weeks after, until delivery)	Appearance of *Lactobacillus* in vaginal flora, the patient achieved pregnancy 3 months later and delivered a healthy infant After the delivery, LF application was discontinued and 1 and 3 months after, no *Lactobacillus* was detected in vaginal discharge cultures	[119]
Randomized, double-blind, placebo-controlled clinical study; *n* = 48 (women of fertile age, 18–50 years, with positive cultures for *Candida* spp., symptomatic acute episodes of VVC and with documented anamnestic history of recurrences confirmed by culture analysis, during the induction phase, being treated with standard antifungal therapy— clotrimazole 100 mg daily for 7 days); Italy	BLF RCX^TM^ (50 mg) plus probiotics (5 × 10^9^ CFU): *L. acidophilus* GLA-14 (LMG S-29159) plus *L. rhamnosus* HN001 (ATCC SD5675) in Respecta^®^ complex (Giellepi S.p.A. Health Science, Lissone, Italy) in p.o. capsule Placebo: identical capsule containing 100 mg maltodextrin; 2 capsules/day, for 5 consecutive days, followed by a further 10 consecutive days at a dosage of 1 capsule/day (acute treatment) During the maintenance phase (6 months following the induction phase), 1 capsule/day for 10 consecutive days per month, in the premenstrual phase (prophylactic treatment) The timeline was related to the consideration that in the premenstrual (luteal) phase, the vagina becomes more vulnerable to the pathogens	Efficacy evaluation based on clinical overall cure rate: vaginal discharge or itching and negative cultures; recurrence rate during the 6-month follow-up period Itching ↓ (at 3 months, women without itching: 70.8% in the Respecta^®^ group vs. 8.3% in the placebo group; at 6 months: 83.3% vs. 0%) Vaginal discharge ↓ (at 3 months women without discharge were 66.7% in Respecta^®^ group vs. 8.3% in placebo group; at 6 months: 70.8% vs. 20.8%) Overall cure rate defined as the absence of any symptoms and yeasts indicated in culture from vaginal swabs ↓ (at 3 months: 66.7% vs. 8.3%; at 6 months: 70.8% vs. 0%) Recurrence rate was defined as the presence of any VVC symptoms and yeasts in culture from vaginal swabs ↓ (at 3 months: 33.3% vs. 91.7%; at 6 months: 29.2% vs. 100%)	[120]
*n* = 34 (women aged 25–45 years with signs and symptoms of acute VVC); Italy	BLF * 4% in vaginal cream, 5 g of cream in the vagina and 2 cm applied on the vulva twice a day for 7 days	Clinical and microscopic examination was performed Response to all the characteristic symptoms of VVC ↑ 27 patients completely recovered, 5 showed a good improvement, and 2 were still suffering from VVC at the end of treatment	[121]
Open-label cohort clinical study; *n* = 7 (pregnant women asymptomatically affected by *Chlamydia * *trachomatis* and showing a high concentration of IL-6 in cervical fluids); Italy	BLF, 20% iron-saturated (Morinaga Milk Ind., Tokyo, Japan), intravaginal, 100 mg every 8 h (daily doses 300 mg/person) for 30 days In vitro test on the anti-chlamydial effect of BLF: human epithelial HeLa-229 cell line was treated with 100 μg/mL BLF	In vivo test: *C. trachomatis* in cervicovaginal smears and IL-6 concentration in the cervical fluid were assessed 6 out of 7 cervical specimens were negative for *C. trachomatis* IL-6 in cervical fluids ↓ Patients achieved pregnancy and delivered at term No maternal and neonatal AEs In vitro test: inhibitory effect of BLF on *C. trachomatis* entry to cells, decrease in IL-6 and IL-8 levels induced by infection with *C. trachomatis*	[86]
One-center, placebo- controlled, cohort clinical study; *n* = 48 (pregnant women at risk of PROM); Egypt	rHLF * 100 mg, p.o. twice/day for 30 days before meals Placebo as control	Patient’s hospital stay → Duration of PROM to delivery → Mode of delivery (vaginal/cesarean section) → Risk of PROM ↓	[122]
Open-label, pilot clinical study; *n* = 21 (26–32 weeks pregnant women, suffering from IDA and abnormal vaginal flora, at risk of preterm delivery); Italy	BLF (Lattoferrina^®^, AG Pharma, Rome, Italy) p.o., 100 mg/day b.i.d. (daily doses 200 mg), before meals, every day for 1 month FeSO_4_ 520 mg in p.o. tablets, as control	Outcomes were clinical characteristics and structure of the vaginal bacterial biota, determined by a culture-based method Normal vaginal microbiota ↑ (vaginal infection disappearance) Cervicovaginal IL-6 (vaginal inflammatory response) ↓ In both groups, cervical length and funneling (in the ultrasound data) did not change at follow-up after 10 and 30 days; pregnancy continued regularly, and all women had term delivery after 37 weeks	[123]
Randomized, open-label study; *n* = 60 (pregnant women undergoing genetic amniocentesis at the 16th gestational week, at risk of inflammation); Italy	BLF (Difesan^®^, Progine Farmaceutici, Firenze, Italy), 300 mg in vaginal tablet, once 4 h or 12 h prior amniocentesis	Amniotic IL-6 ↓	[124]
		Decreased levels of amniotic pro-inflammatory mediators: IL-9, IL-15, IFN-γ, IP-10, TNF-α, IL-1α, MCP-3, IL-2RA, IL-12p40, IFN-α2, IL-2, IL-4, eotaxin, PDGF-BB, RANTES, IL-18, MIF ↓ Increased levels of amniotic anti-inflammatory mediators: IL-17, FGF-b, G-CSF, GM-CSF, MCP-1, IL-3, SDF-1α ↑	[125]
	BLF (Difesan^®^, Progine Farmaceutici, Firenze, Italy), 300 mg in a vaginal tablet, once every 4 h or 12 h prior to amniocentesis In vitro test on the antioxidant effect of LTF: human monocytic U937 cell line was treated with 50 μg/mL LTF ^#^ for 4 h or 12 h	Decreased oxidative stress in vivo: Amniotic TBARS concentration ↓ Amniotic TAS ↑ Decreased oxidative stress in vitro: TBARS concentration ↓	[126]
Prospective, randomized study; *n* = 111 (pregnant women undergoing genetic amniocentesis at the 16–18th gestational week, at risk of inflammation); Italy	BLF (Difesan^®^, Progine Farmaceutici, Firenze, Italy), 300 mg in vaginal tablet, once every 4 h prior to amniocentesis	Regulation of the inflammatory markers in the amniotic fluid: PGE2, MMP-9 and TIMP-1 (inhibitor of MMP-1) ↓ MMP-2 ↑ TIMP-2 (inhibitor of MMP-2) →	[127]

AEs—adverse events; BLF—bovine lactoferrin; BV—bacterial vaginosis; CFU—colony forming units; FGFb—basic fibroblast growth factor; G-CSF—granulocyte colony-stimulating factor; GM-CSF—granulocyte-macrophage colony-stimulating factor; IFN—interferon; IL—interleukin; IP-10—interferon inducible protein; LF—lactoferrin; MCP-1—monocyte chemoattractant protein-1; MIF—macrophage migration inhibitory factor; MMP-2—matrix metalloproteinase-2; MMP-9—matrix metalloproteinase-9; OSI—oxidative stress index; PDGF—platelet-derived growth factor; PGE2—prostaglandin E2; PROM—premature rupture of membrane qPCR—quantitative polymerase chain reaction; RANTES—regulated on activation normal T cells expressed and secreted; rHLF—recombinant human LF; SDF-1α—stromal cell-derived factor 1α; TAS—total antioxidant status (expressed in Trolox equivalents or OSI); TBARS—thiobarbituric acid reactive substances (expressed as malondialdehyde—MDA equivalents); TIMP-1—tissue inhibitor of metalloproteinase-1; TIMP-2—tissue inhibitor of metalloproteinase; TNFα—tumor necrosis factor α; VVC—vulvovaginal candidiasis; * the authors did not identify what kind of LF preparation was used or only an abstract was available. ↑ increase, ↓decrease, → no change.

## Data Availability

No new data were created or analyzed in this study. Data sharing is not applicable to this article.
